# Pharmacological treatment of post-traumatic stress disorder- an audit of Cardiff Health access practice using a pharmacological prescribing algorithm

**DOI:** 10.1192/bjo.2021.820

**Published:** 2021-06-18

**Authors:** Amy Baker, Jonathan Bisson

**Affiliations:** Cardiff University

## Abstract

**Background:**

Post-Traumatic Stress Disorder (PTSD) is a mental health disorder characterised by symptoms of re-experiencing, avoidance and hyperarousal that may develop after exposure to a traumatising event. The prevalence of PTSD within the refugee population is ten times higher than in the general population. This audit was carried out in Cardiff Health Access Practice (CHAP) which is the main provider of primary health care for refugees and asylum seekers who are sent to Cardiff. The main objective of this audit was to evaluate current PTSD prescribing practice for patients presenting to Cardiff Health Access Practice (CHAP) against a pharmacological prescribing algorithm which has been developed for the Cardiff and Vale Traumatic Stress Service based on NICE and International Society for Traumatic Stress Studies guidelines

**Method:**

A retrospective audit of patients with PTSD seen in the last 12 months at CHAP. Data were collected from patient notes and information on age, sex, trauma, comorbidities and medication dose was collated and analysed using SPSS statistics.

**Result:**

130 patients with PTSD were identified and their medications assessed for the audit. The mean age of these patients was 33 years and there was a 1.5:1 male to female ratio. Of the 130 patients only 10 were initiated on a first line medication, 117 were started on a fourth line medication. No patients were prescribed either the second- or third-line medications.

**Conclusion:**

The low rates of compliance with the All Wales Pharmacological PTSD pharmacological prescribing algorithm are disappointing although not unexpected as it has yet to be fully introduced to the service. Following discussion of the results and teaching about the algorithm with clinicians in Cardiff Health Access Practice rates of evidence-based prescribing should improve. This audit focuses on a patient group (refugee and asylum seekers) which has been identified as a priority group by the Welsh Government. Through further implementation of this algorithm there should be improved evidence-based prescribing and continuity of care for refugees

